# P-2123. Splenectomy in Cancer Patients. Is It Associated with a High Risk of Infection?

**DOI:** 10.1093/ofid/ofaf695.2287

**Published:** 2026-01-11

**Authors:** Pamela Alatorre Fernandez, Beda Islas Muñoz, Patricia Volkow Fernandez, Roan Villagran Ruiz, Patricia Cornejo Juarez

**Affiliations:** Instituto Nacional de Cancerología, Mexico City, Distrito Federal, Mexico; Instituto Nacional de Cancerologia, Mexico, Distrito Federal, Mexico; Instituto Nacional de Cancerologia, Mexico, Distrito Federal, Mexico; Instituto Nacional de Cancerologia, Mexico, Distrito Federal, Mexico; Instituto Nacional de Cancerología, Mexico City, Distrito Federal, Mexico

## Abstract

**Background:**

Splenectomy is considered to increase the risk of infection. To mitigate this risk, immunization and antibiotic prophylaxis are recommended.

**Table 1.**
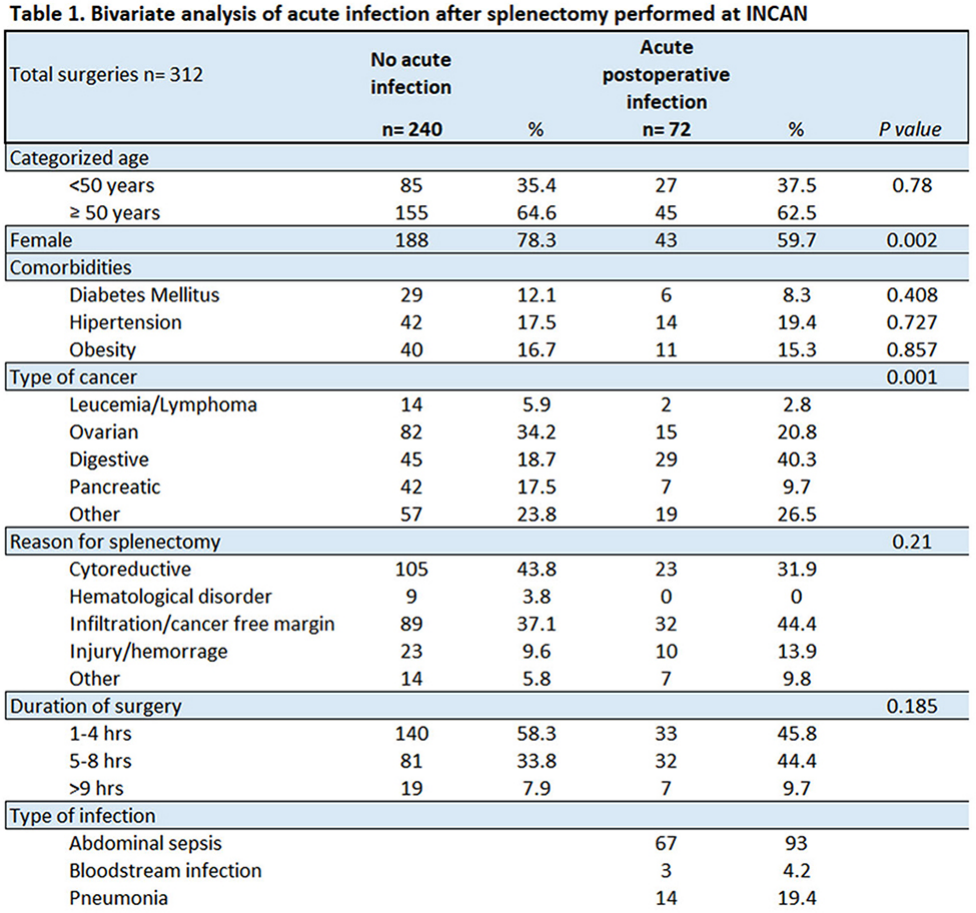
Bivariate analysis of acute infection after splenectomy performed at a cancer referral center

**Graph 1.**
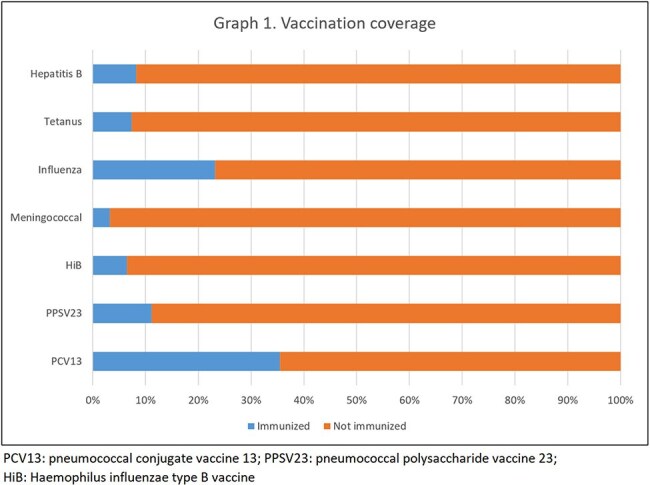
Vaccination coverage

**Methods:**

A retrospective review of the medical records of 356 patients with cancer treated at a Referral Center in Mexico (INCan) who were splenectomized between 2010 and 2024 was performed. Demographic characteristics, oncological diagnosis, reason for splenectomy, acute infections, adherence to vaccination recommendations, overwhelming post-splenectomy infection (OPSI), and status at the end of follow-up were recorded.

**Table 2.**
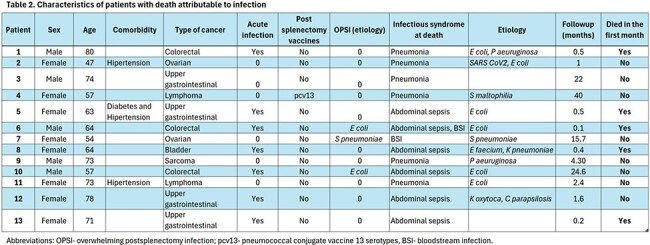
Characteristics of patients with death attributable to infection

**Results:**

356 cases: 249 (70%) women with a median age of 54 years (IQR 44-64); The primary types of cancer were ovarian (n = 98, 27.5%), digestive (n = 81, 22.0%), pancreatic (n = 51, 14.3%), and lymphoma (n = 30, 8.4%). The main reasons for splenectomy were cytoreductive surgery (n = 135, 38%), infiltration/tumor-free surgical margins (n = 132, 37%), and injury/hemorrhage (n = 35, 10%).

Bivariate analysis including patients operated at INCan (n = 312, 87.6%), compared 240 cases that did not develop an acute infection with 72 who did. The proportion of men was 29/81 (35.8%) compared to 42/231 women (18.2%) (p = 0.002). The type of cancer (solid vs. hematologic) did not significantly affect the risk of postoperative infection (p = 0.264). Other data are presented in Table 1. The most common infection was abdominal sepsis (n = 67, 93%), gram-negatives were isolated in 22 (42%) and polymicrobial in 20 (28%). The vaccination coverage after splenectomy is presented in Graph 1.

The median follow-up was 32 months (IQR 11.3-62.9). Thirteen patients (3.6%) experienced death attributable to infection, of whom five died within the first 30 days. These cases are presented in Table 2. Three patients developed OPSI, two due to *E. coli* and one due to *S. pneumoniae*; the cumulative incidence was 0.84/100 patients. No other events involving encapsulated microorganisms were recorded.

**Conclusion:**

Even in our patients immunocompromised by cancer and, despite our low vaccination coverage, the incidence of infections due to encapsulated microorganisms and death attributable to infection was lower than previous reports. So standardized infection prevention, surveillance, and vaccination are recommended for all cancer patients, including splenectomized.

**Disclosures:**

All Authors: No reported disclosures

